# Microbiome-metabolome analysis reveals alterations in the composition and metabolism of caecal microbiota and metabolites with dietary Enteromorpha polysaccharide and Yeast glycoprotein in chickens

**DOI:** 10.3389/fimmu.2022.996897

**Published:** 2022-10-13

**Authors:** Teketay Wassie, Bei Cheng, Tiantian Zhou, Lumin Gao, Zhuang Lu, Chunyan Xie, Xin Wu

**Affiliations:** ^1^ Key Laboratory of Agro-ecological Processes in Subtropical Region, Institute of Subtropical Agriculture, Chinese Academy of Sciences; National Engineering Laboratory for Pollution Control and Waste Utilization in Livestock and Poultry Production, Hunan Provincial Engineering Research Center for Healthy Livestock and Poultry Production, Changsha, China; ^2^ College of Bioscience and Biotechnology, Hunan Agricultural University, Changsha, China; ^3^ Tianjin Institute of Industrial Biotechnology, Chinese Academy of Sciences, Tianjin, China

**Keywords:** microbiota, metabolite, entromorpha polysaccharide, yeast glycoprotein, immunity

## Abstract

The intestinal microbiome is responsible for the fermentation of complex carbohydrates and orchestrates the immune system through gut microbiota-derived metabolites. In our previous study, we reported that supplementation of *Enteromorpha* polysaccharide (EP) and yeast glycoprotein (YG) in combination synergistically improved antioxidant activities, serum lipid profile, and fatty acid metabolism in chicken. However, the mechanism of action of these polysaccharides remains elusive. The present study used an integrated 16S-rRNA sequencing technology and untargeted metabolomics technique to reveal the mechanism of action of EP+YG supplementation in broiler chickens fed basal diet or diets supplemented with EP+YG (200mg/kg EP + 200mg/kg YG). The results showed that EP+YG supplementation altered the overall structure of caecal microbiota as evidenced by β diversities analysis. Besides, EP+YG supplementation changed the microbiota composition by altering the community profile at the phylum and genus levels. Furthermore, Spearman correlation analysis indicated a significant correlation between altered microbiota genera vs serum cytokine levels and microbiota genera vs volatile fatty acids production. Predicted functional analysis showed that EP+YG supplementation significantly enriched amino acid metabolism, nucleotide metabolism, glycan biosynthesis and metabolism, energy metabolism, and carbohydrate metabolism. Metabolomics analysis confirmed that EP+YG supplementation modulates a myriad of caecal metabolites by increasing some metabolites, including pyruvic acid, pyridoxine, spermidine, spermine, and dopamine, and decreasing metabolites related to lipid metabolisms such as malonic acid, oleic acid, and docosahexaenoic acid. The quantitative enrichment analysis results further showed that glycolysis/gluconeogenesis, citric acid cycle, tyrosine metabolism, glycine, serine, and threonine metabolism, and cysteine and methionine metabolism were the most important enriched pathways identified with enrichment ratio >11, whereas, fatty acid biosynthesis and biosynthesis of unsaturated fatty acids pathways were suppressed. Together, the 16S-rRNA and untargeted metabolomics results uncovered that EP+YG supplementation modulates intestinal microbiota and their metabolites, thereby influencing the important metabolism pathways, suggesting a potential feed additive.

## 1 Introduction

Dietary fibers have been recognize to play an important role in improving health and energy hemostasis (1). However, humans and animals have no enzymes to digest complex carbohydrates, while gut microbes could digest dietary fibers and produce useful metabolites (2). Therefore, the intestinal microbiota is essential for the host to utilize the complex carbohydrate, while the host provides habitat for them (3). The chicken gut particularly the caecum is colonized by tremendous metabolically active microbes (4), which are involved in different physiological activities such as immune response and energy homeostasis (5). Apart from their direct effect, gut microbiota produces short-chain volatile fatty acids (SCFAs) (6) that regulate intestinal immune response and barrier function (7, 8).

In addition to SCFA, other microbiota-derived metabolites, such as bile acids, vitamins, amino acids, serotonin and polyamines, trimethylamine, and hypoxanthine serve as signaling molecules and involve in metabolism and immune regulation that influence biological functions (9). Thus, alterations of these metabolites may be associated with the manifestation of disease in the host. Therefore, a comprehensive analysis of metabolites is essential to identify their role in immunity and metabolism. Metabolomics is an emerging technology that analysis the profiling of metabolites of a given sample to identify their biomarker role in health.

Mounting evidence has shown that dietary intervention modulates the host intestinal microbiota composition, diversity, and function, thereby altering health and energy homeostasis (10). Recent studies highlighted that algae-derived and yeast-derived polysaccharides could modulate gut microbiota and microbial-derived metabolites in different species of animals, which in turn enhance host immunity and metabolism (11, 12).

As an effective antibiotic alternative, polysaccharides extracted from *Enteromorpha prolifera* (EP) alleviated LPS-induced intestinal inflammation, modulated the gut microbiota (13), stimulated SCFA production (14), improved growth performance, egg production, antioxidant activity, immune response, and fatty acid and amino acid metabolism in chickens (15, 16).

Yeast glycoprotein (YG) is the yeast cell wall extract that possesses polysaccharides (β-glucan and mannan oligosaccharide) and protein. Dietary yeast glycoprotein supplementation promotes growth performance and improves gut health in weaned piglets (17). Furthermore, mannan oligosaccharide (MOS) supplementation has been shown to improve growth performance (18, 19), antioxidant activity (20), gut health and alleviates intestinal inflammation in chickens (20–22). Similarly, previous reports have shown that β-glucan inclusion altered the gut microbiota in mouse models (23, 24) and older hens (25), and improved growth performance (26), gut health (27), immunity (28, 29) and mitigate intestinal inflammation (26), thereby used as an alternative to antibiotics (30).

Therefore, it is imperative to identify the microbiota responsible for the fermentation of these polysaccharides and their metabolites to develop an effective antibiotic alternative. Given the above information, we assessed the impact of the combined EP and YG polysaccharide supplementation on the gut microbiome and metabolites and their association with immune responses using 16S-rRNA sequencing and untargeted metabolomics.

## 2 Materials and methods

### 2.1 Source of *Enteromorpha* polysaccharide

The polysaccharide was extracted from marine algae *Enteromorpha prolifera* and provided by Qingdao Seawin Biotechnology Group Co., Ltd. (Qingdao, China). The detailed extraction methods and composition of this polysaccharide were described in our previous studies (14, 15).

### 2.2 Bird management

The experimental design and procedures used in this study were reviewed and approved by the Animal Care and Use Committee of the Institute of Subtropical Agriculture, Chinese Academy of Sciences (2021-0036A). The animal experiments and sample collection strictly followed the relevant guidelines.

A total of 200 healthy one-day-old male Ross-308 broiler chickens were obtained from a local hatchery and housed in wire cages (10 birds/cage) raised in a room where temperature and ventilation were controlled. Briefly, the chickens were kept in a room with 23-h of light and 1-h darkness. The room temperature was kept at about 32°C for 3 d and gradually reduced by 1°C every other day until the temperature reached 24°C, and then maintaining this temperature according to our previous studies (15). Feed and water were provided *ad libitum*. The basal diet was formulated following the guidelines for Ross broiler chickens (14) (**Supplementary Table 1**).

### 2.3 Diet and experimental design

The experiment was performed in a complete randomized design, and 200 one-day-old male Ross-308 broiler chickens were randomly divided into two treatment groups with ten replications of ten chickens per replication. The first group was fed a basal diet (control group), and the second group was fed a basal diet supplemented with 400 mg/kg diet (EP + YG in 1:1 ratio) according to our previous study (14). The experiment lasted for 42 days.

### 2.4 Sample collection

At the end of the experiment (d 42), one chicken from each replication (n=10/treatment) close to the average body weight of the group was selected, blood samples were collected and then sera were separated by centrifuging at 4000 rpm for 10 min at 4°C and stored at -20 °C for subsequent analysis. The chickens were then humanely killed by cervical dislocation, and caecal content was isolated, immediately frozen in liquid nitrogen, and stored at -80°C until analysis.

### 2.5 Cytokine analysis

The serum cytokine contents of interleukin-1β (IL-1β), IL-2, IL-6, IL-10, tumor necrosis alpha (TNF-α), and interferon-gamma (INF-γ) were performed using ELISA kits (Shanghai Kexin Biotech Co., Ltd, Shanghai, China).

### 2.6 Short-chain volatile fatty acid analysis

The short‐chain volatile fatty acids (acetate, butyrate, propionate, iso-butyrate, valerate, and iso-valerate) were determined from caecal digesta samples using the Agilent 6,890 gas chromatography (Agilent Technologies, Inc, Palo Alto, CA) according to the previous study (31).

### 2.7 16S-rRNA amplicon sequencing

#### 2.7.1 Genomic DNA extraction and 16S-rRNA gene sequencing

The microbial DNA extraction from caecal content was performed using the E.Z.N.A. Stool DNA Kit (D4015, Omega, Norcross GA, USA) and the quality and concentration of DNA were assessed using NanoDrop 2000 spectrophotometer (Thermo Scientific, Waltham, MA, USA). Following extraction, PCR was used to amplify the V3-V4 region of the bacterial 16S-rRNA gene using the primers (F: 5’-ACTCCTACGGGAGGCAGCAG-3’; R: 5’-GGACTACHVGGGTWTCTAAT-3’). The PCR product was then run on 2% agarose gel, and then excised and purified using Qiagen gel extraction kit (Qiagen, Germany) and quantified using Quantus™ Fluorometer (Promega, Madison, USA).

TruSeq^®^ DNA PCR-Free Sample Preparation Kit (Illumina, San Diego, USA) was used to prepare DNA library and then evaluated on the Qubit^®^ 2.0 Fluorometer and Agilent Bioanalyzer system (Thermo Fisher Scientific, Waltham, MA, USA). The DNA sequencing was performed on an Illumina HiSeq platform by Novogene Bioinformatics Technology Co., Ltd. (Beijing, China).

#### 2.7.2 16S-rRNA data analysis

Paired-end reads from the original DNA fragments were merged by FLASH and resulting labels were assigned to the Operational Taxonomic Units (OTUs) with a threshold value of 97% using UPARSE (http://drive5.com/uparse) (32). The taxonomic information of the representative sequence was annotated through the Silva Database (http://www.arb-silva.de/) with Mothur algorithm (33). The species diversity (α-diversity) Chao, Shannon, and Simpson indices were estimated using QIIME2 (34). Principal coordinate analysis (PCoA) was performed using UniFrac distance metrics. Analysis of similarity (ANOSIM) was used to test the statistical differences among the groups. Linear discriminant analysis (LDA) effect size (LEfSe) was performed to reveal the difference in the bacterial communities across the treatments using the non-parametric factorial Kruskal-Wallis test with an alpha value of 0.05 and LDA score of 4. In addition, the relative abundance of dominant bacteria at the phylum and genus levels was also analyzed. Spearman correlation was used to investigate the association between gut microbiota and SCFA production.

### 2.8 Metabolomics analyses

#### 2.8.1 Sample preparation

The caecal metabolites were determined by a commercial service in LC-Bio (Hangzhou, Zhejiang, China). The collected samples (8 samples from each group) were thawed on ice, and metabolites were extracted with 50% methanol. Briefly, 20 μL of the sample was extracted with 120 μL of precooled 50% methanol, vortexed for 1 min, and incubated at room temperature for 10 min; the extraction mixture was then stored overnight at -20°C. After centrifugation at 4,000 g for 20 min, the supernatants were transferred into new 96-well plates. The samples were stored at -80°C before the LC-MS analysis. In addition, pooled QC samples were also prepared by combining 10 μL of each

#### 2.8.2 Metabolite analysis

All samples were acquired by the LC-MS system and chromatographically separated using a Thermo Scientific UltiMate 3000 HPLC and reversed phase-separated using ACQUITY UPLC BEH C18 column (100mm*2.1mm, 1.8µm, Waters, UK). The column oven was maintained at 35°C. The flow rate was 0.4 ml/min and the mobile phase consisted of solvent A (water, 0.1% formic acid) and solvent B (Acetonitrile, 0.1% formic acid). Gradient elution conditions were set as follows: 0~0.5 min, 5% B; 0.5~7 min, 5% to 100% B; 7~8 min, 100% B; 8~8.1 min, 100% to 5% B; 8.1~10 min, 5%B. The injection volume for each sample was 4 µl.

A high-resolution tandem mass spectrometer Q-Exactive (Thermo Scientific) was used to detect metabolites eluted from the column. The Q-Exactive was operated in both positive and negative ion modes. The curtain gas was set at 30 PSI, Ion source gas1 was set at 60 PSI, Ion source gas2 was set at 60 PSI, an interface heater temperature was 650 °C and Ionspray voltage floating was 5000 V for positive ion mode and -4500V for negative ion mode. Precursor spectra (70-1050 m/z) were collected at 70,000 resolution to hit an AGC target of 3e6. The maximum inject time was set to 100 ms. A top 3 configuration to acquire data was set in DDA mode. Fragment spectra were collected at 17,500 resolution to hit an AGC target of 1e5 with a maximum inject time of 80 ms. To evaluate the stability of the LC-MS during the whole acquisition, a quality control sample (pool of all samples) was acquired after every 10 samples.

#### 2.8.3 Data processing and analysis

LC-MS raw data files were converted into mzXML format and then processed by the XCMS, CAMERA, and metaX (35) toolbox implemented with the R software and then, the peak was detected, and extracted, aligned, and integrated by XCMS. The online Kyoto Encyclopedia of Genes and Genomes (KEGG) database was used to annotate the metabolites by matching the exact molecular mass data (m/z) of samples with those from the database. Each parameter was optimized individually, and the manual extraction of arbitrary mass chromatogram peaks was used to verify the accuracy of the results. SIMCA-P (16.0.2, Sartorius Stedim Data Analytics AB, Umea, Sweden) was used to perform the principal component analysis (PCoA) and partial least squares discriminant analysis (PLS-DA). Finally, the t-test was used to determine the effective differential metabolites and the variables with significant differences between the groups. To screen significantly different metabolic markers, univariate statistical analysis with the criteria of variable importance in the projection (VIP)>1 and the fold change of metabolites less than 0.5 or more than 2, coupling with P-value <0.05 was used, which was visualized by volcano plot and heatmap. The KEGG database was used to determine the relevant significant changed metabolism pathway.

### 2.9 Statistical analysis

The serum cytokine and caecal volatile fatty acid production data were analyzed using the statically analytical software (SAS 9.1 Institute, Inc., Cary, NC, USA). The normality and homoscedasticity of the data variance were checked using the Shapiro-Wilk test and Levene’s test, respectively. Then, data were subjected to independent t-test and statistically significant was considered when *P<*0.05. The 16S-rRNA sequencing data were analyzed using https://www.omicstudio.cn/tool. The metabolomics data were analyzed using online tool www.metaboanalyst.com.

## 3 Results

### 3.1 EP+YG alters serum cytokine profile

**Figure 1:**
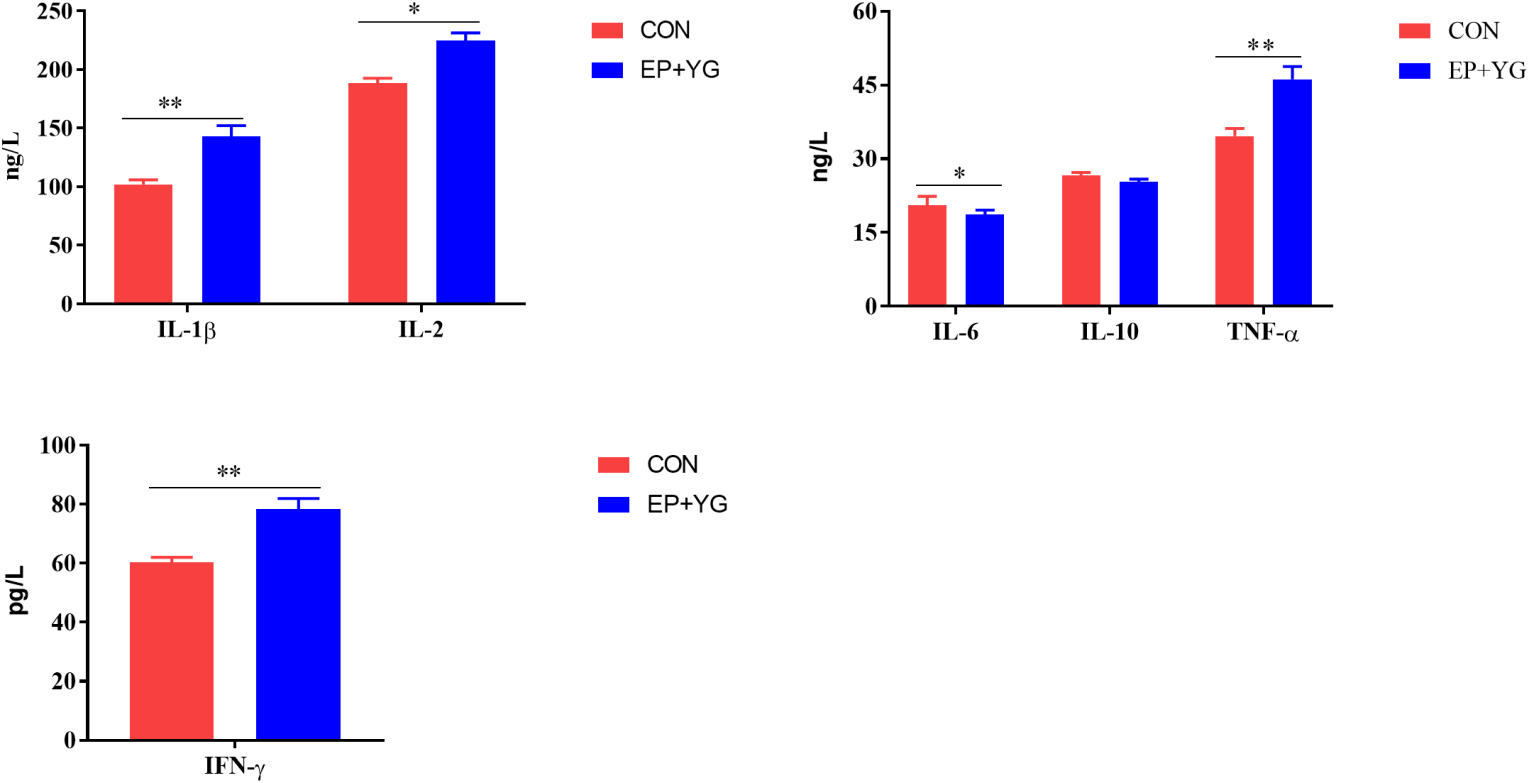
The effect of dietary EP+YG inclusion on the serum cytokine levels of broiler chickens. Data are presented as mean ± SEM, n = 8. *P < 0.05 and **P < 0.01. IL-1β, Interleukin 1 beta; IL-2, Interleukin 2; IL-6, Interleukin 6; IL-10, Interleukin 10; TNF-α, Tumor necrosis factor-alpha; IFN-interferon-gamma.

To assess whether EP+YG supplementation regulates immune response, we examined the serum cytokine contents using ELISA. The results (**Figure 1**) demonstrated that the serum IL-1β, IL-2, TNF-α, and IFN-γ levels were significantly (*P*<0.05) higher while the IL-6 content was lower in the EP+YG than in the control group. However, a significant treatment effect on IL-10 content was not observed.

### 3.2 EP+YG stimulates caecal volatile fatty acid production

**Figure 2:**
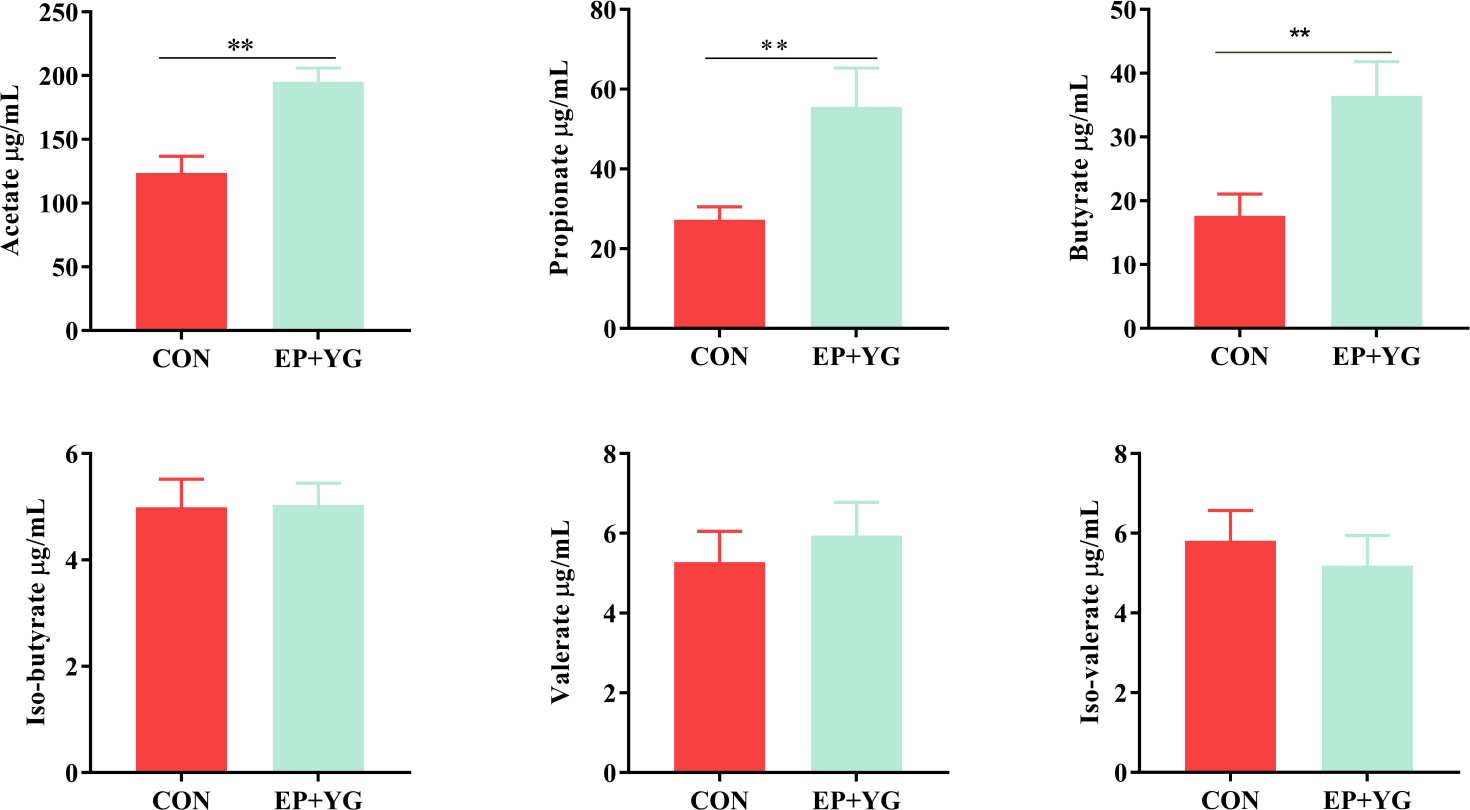
Effect of EP+YG supplementation on caecal content SCFA concentration in broiler chickens. n=8, ** indicate significant differences at *P* < 0.05 and *P* < 0.01, respectively.

To assess whether EP+YG supplementation influences SCFAs production, we measured SCFA from caecal content using Gas chromatography. The results (**Figure 2**) demonstrated that chickens fed a diet containing EP+YG had significantly higher (*P*<0.05) acetate, butyrate, and propionate content than chickens in the control group. In iso-butyrate, valerate, and iso-valerate contents, we did not observe a significant difference between the treatment groups.

### 3.3 EP+YG supplementation alters the caecal microbiota structure

**Figure 3:**
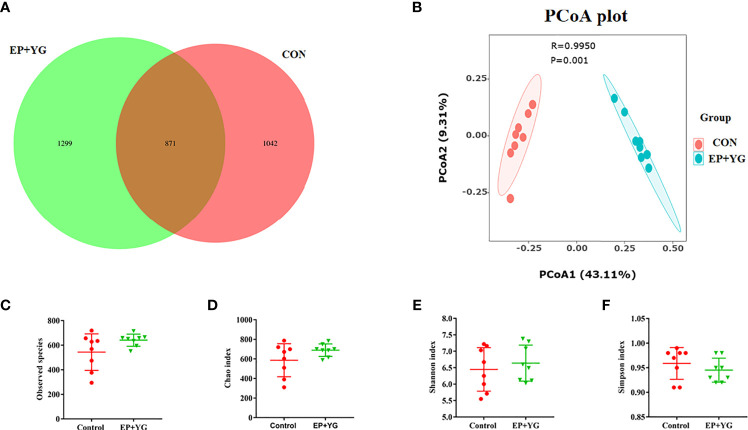
The Venn diagram, alpha and beta diversity analysis of caecal microbiota of treatment and control group chickens (n=8). **(A)** Venn diagram shows the unique and shared OTUs between treatment groups; **(B)** principal coordinate analysis (PCoA) generated using Bray-Curtis dissimilarity approach **(C)** observed species, **(D)** Chao index, **(E)** Shannon index, **(F)** Simpson index. Statistical significance was assessed using the Kruskal-Wallis test. CON (fed basal diet); EP+YG (supplemented with 200 mg EP and 200 mg YG/kg diet in combination).

To determine the impact of supplementation of a mixture of EP and YG on gut microbiota, broiler chickens were fed for 42 days. The caecal contents were collected from the control and treatment groups and the microbial communities’ profiles were examined. An average of 83083 raw data per sample were retrieved and 73119 clean data were obtained after quality trimming and chimera checking. The Venn diagram in (**Figure 3A**) identified 1299 and 1042 unique OTUs in EP+YG and control groups, respectively, and both groups shared 871 OTUs.

The alpha diversity results (**Figures 3C–F**) showed that caecal bacteria richness and diversity appeared to be unchanged by EP+YG supplementation (*P*<0.05). To further insight into the change in bacterial community structure among the treatment groups, β diversity analysis was employed by Principal Coordinate Analysis (PCoA) using the Bray-Curtis index. The result revealed a significantly distinct clustering between the two groups (*P*<0.05) (**Figure 3B**).

### 3.4 EP+YG supplementation changes caecal microbial community profile

**Figure 4:**
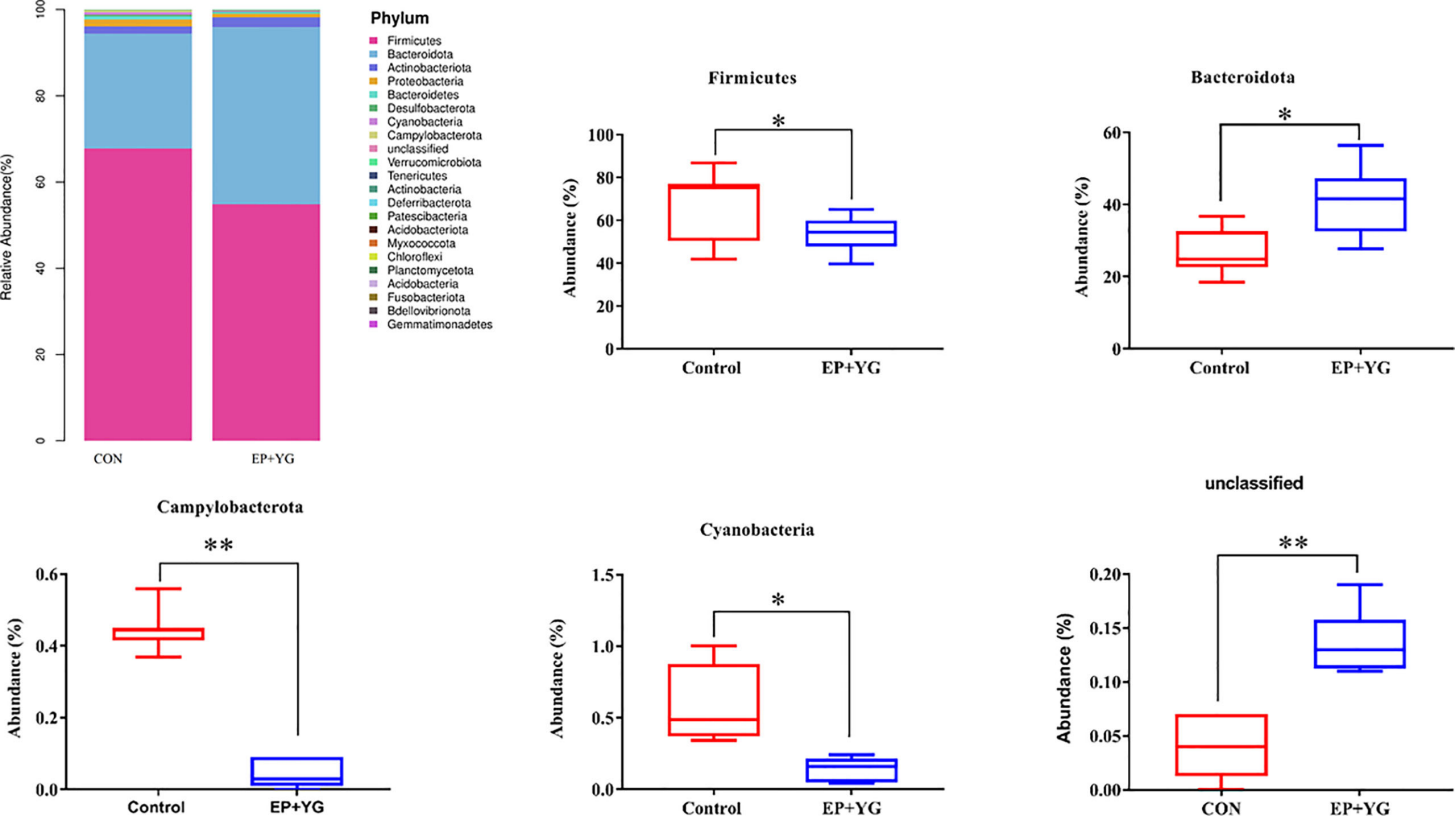
Relative abundances of microbial communities at the phylum level in the caecal content of EP+YG treated and control broiler chickens (n=8). Major phyla for each treatment group and a significant phylum between treatment groups are presented. * *p* < 0.05, ** *p* < 0.01. CON (fed basal diet); EP+YG (supplemented with 200 mg EP plus 200 mg YG/kg).

Next, we examined whether EP+YG supplementation alters the gut microbiota community profile by classifying the observed OTUs into different taxonomic levels. The taxonomic analysis uncovered a distinct pattern in caecal microbiota profiles among treatment groups. At the phylum level (**Figure 4**), the caecal microbiota were primarily dominated by *Firmicutes* and *Bacteroidota*, which together represented an average of more than 90% of the bacterial community. We observed a significant increment (*P*< 0.05) in the abundance of phyla *Bacteroidota* and *unclassified bacteria* and a decrement (*P*< 0.05) in phyla *Firmicutes*, *Cyanobacteria*, and *Campylobacterota* in the EP+YG group compared with the control group (**Figure 4**).

**Figure 5:**
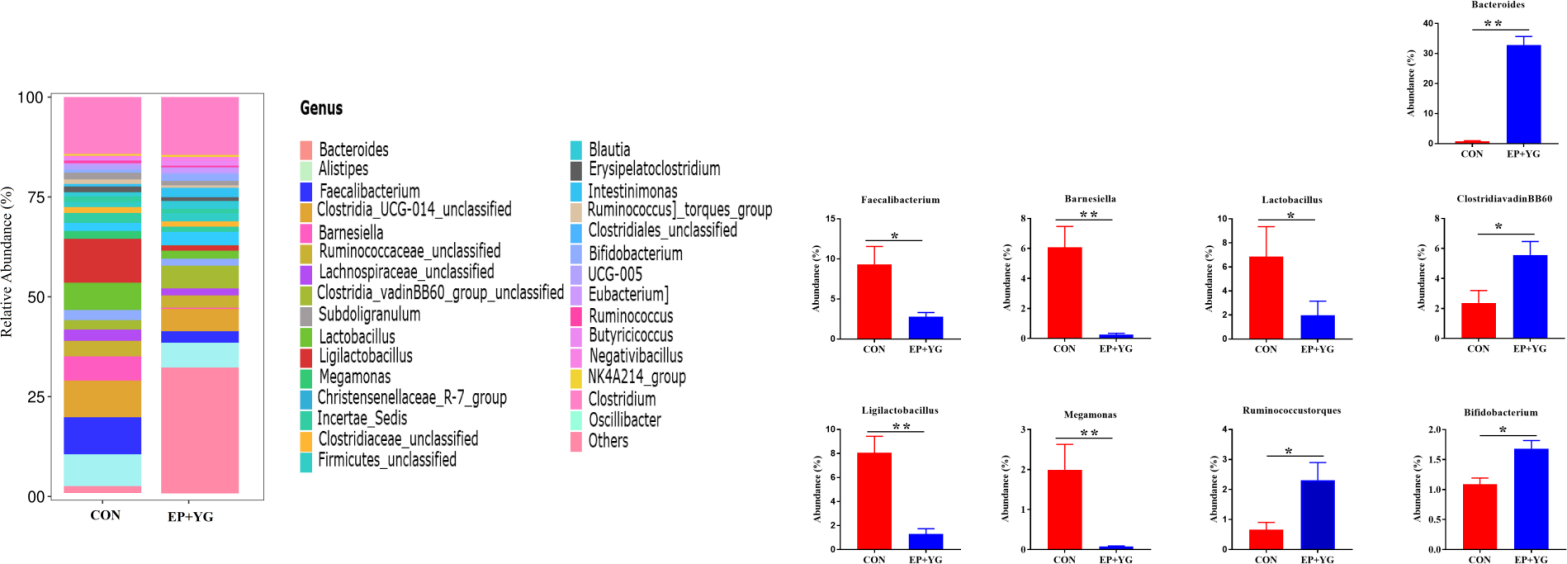
Relative abundances of bacterial communities at the genus levels in the caecal content of EP+YG treated and control group chickens (n=8). The top 30 taxa for each treatment group and significant taxa between treatment groups are presented, * *p* < 0.05, ** *p* < 0.01. CON (Fed basal diet); EP+YG (supplemented with 200 mg EP and 200 mg YG/kg diet in combination).

At the genus level (**Figure 5**), EP+YG treatment appeared to increase *Bacteroides, Clostridia_vadinBB60_group_unclassified*, *Ruminococcus]_torques_group* and *Bifidobacterium* and decreased *Faecalibacterium*, *Barnesiella*, *Lactobacillus*, *Ligilactobacillus*, and *Megamonas*.

### 3.5 EP+YG supplementation causes selective enrichment of microbial communities

**Figure 6:**
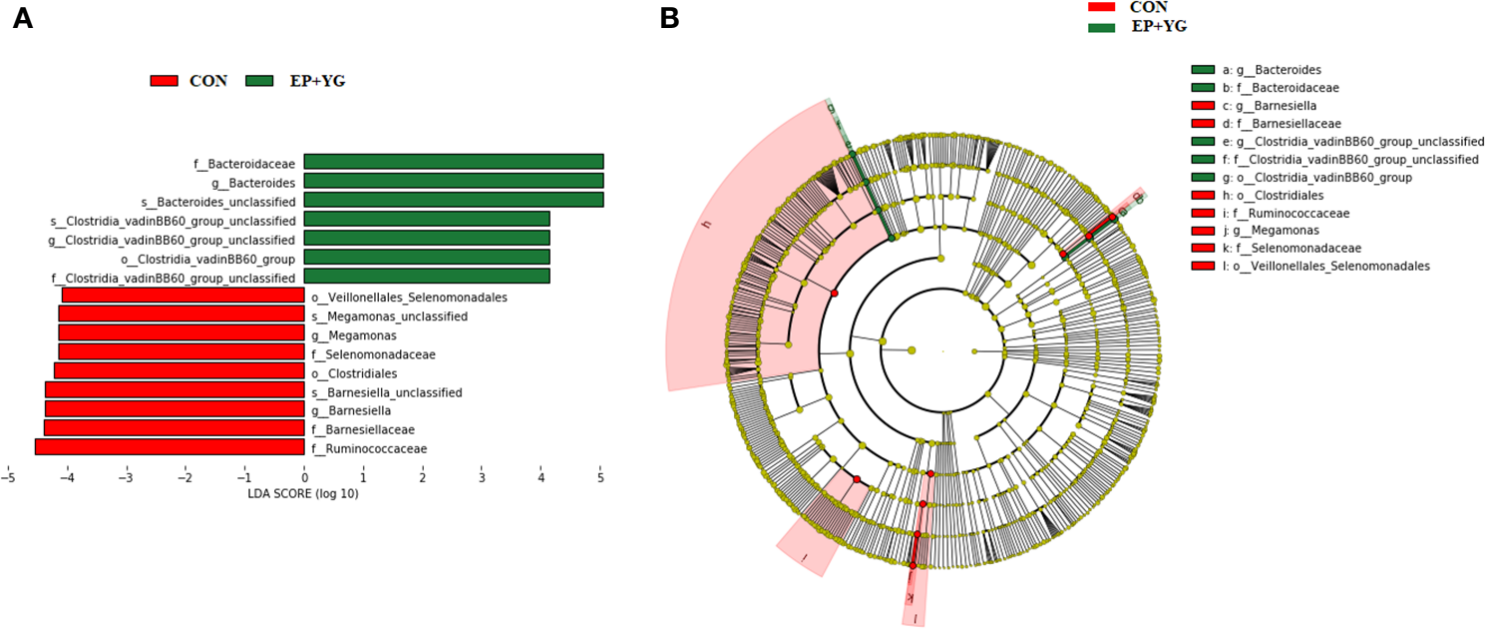
Linear discriminate analysis (LDL) effect size (LEFSe) analysis identified microbial taxa between EP+YG (green) and control (red) group. **(A)** Histogram plot from LEfSe analysis that presents the LDA scores of microbial taxa whose abundance showed significant differences between control and EP supplemented group broiler chickens (LDA score > 4). The length of the bar column represents the LDA score. **(B)** In the cladogram, circles radiating from the inner side to the outer side represent the differences in the relative abundance of taxa from phylum to genus level between EP and control groups.

Based on the above results, we further did a Linear discriminant analysis (LDA) effect size (LEfSe) analysis to identify the enriched microbial community from phylum to species among the treatment groups with an LDA score >4 (**Figure 6**). Dietary EP+YG supplementation selectively enriched the abundance of genera *Bacteroides* and *Clostridia_vadinBB60_group* while genera *Barnesiella* and *Megamonas* were enriched in the control group.

### 3.6 EP+YG supplementation affects the predictive function of gut microbiota

**Figure 7:**
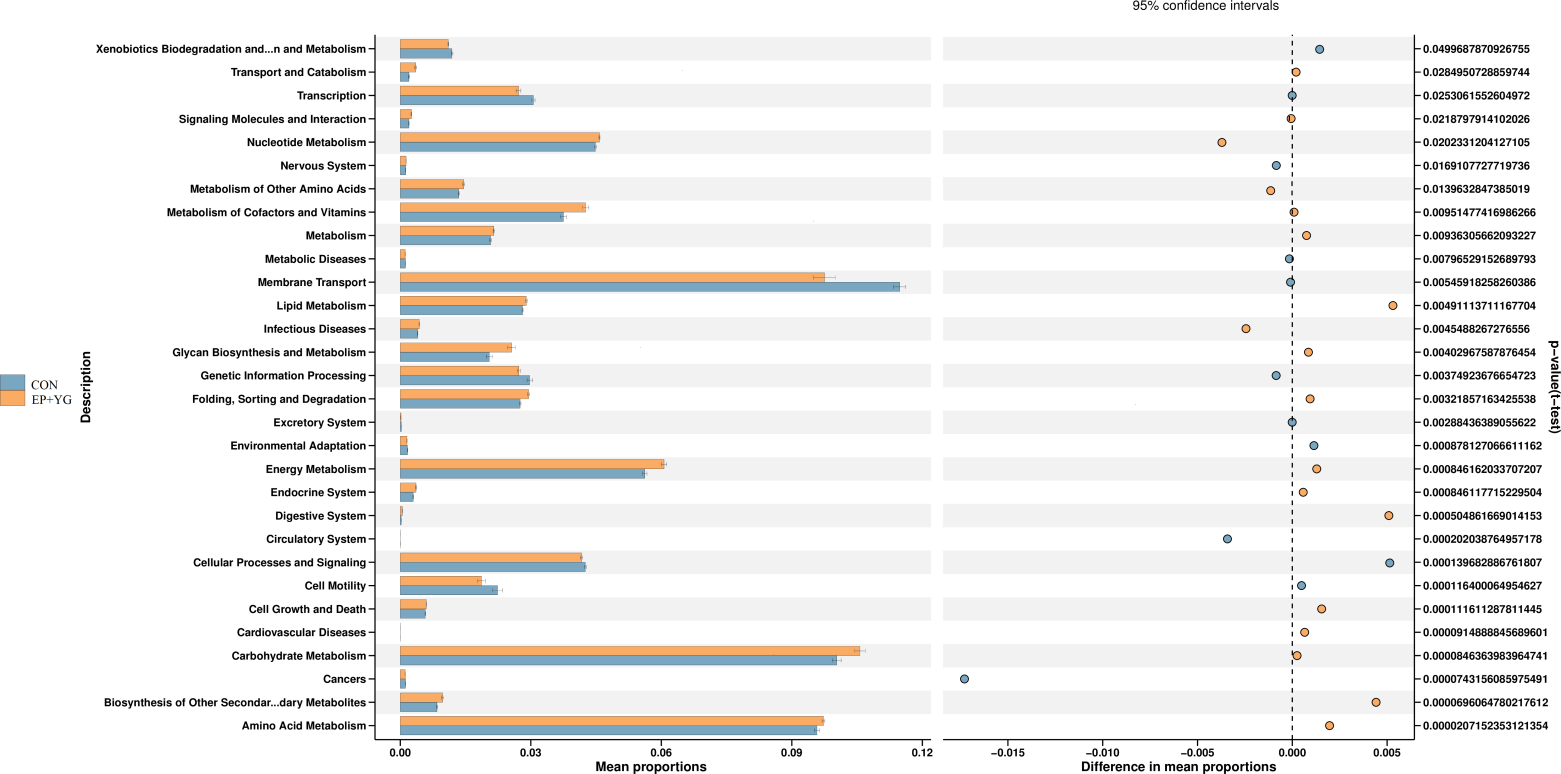
Predictive functional analysis of caecal microbiota in the EP+YG treated and control broiler chickens. Predictive functional analysis was conducted using PICRUSt at the second level of the KEGG pathway. CON (fed basal diet); EP+YG (supplemented with 200 mg EP and 200 mg YG/kg diet in combination).

We next predict the function of the observed microbiota using KEGG pathways to identify the functional pathways affected by EP+YG treatment. The prediction analysis identified 30 functional pathways between EP+YG vs CON groups (**Figure 7**). Among the 30 pathways identified, EP+YG supplementation significantly enriched transport and catabolism, signal molecule and interaction, nucleotide metabolism, metabolism of other amino acids, metabolism of cofactors and vitamins, metabolism, glycan biosynthesis and metabolism, energy metabolism, digestive system, carbohydrate metabolism, biosynthesis of other secondary metabolites and amino acid metabolism pathways, while suppressed xenobiotics degradation and metabolism, transcription, membrane transport, genetic information processing, cellular process, and signaling and cell motility pathways.

### 3.7 Correlation between microbiota and cytokine content

**Figure 8:**
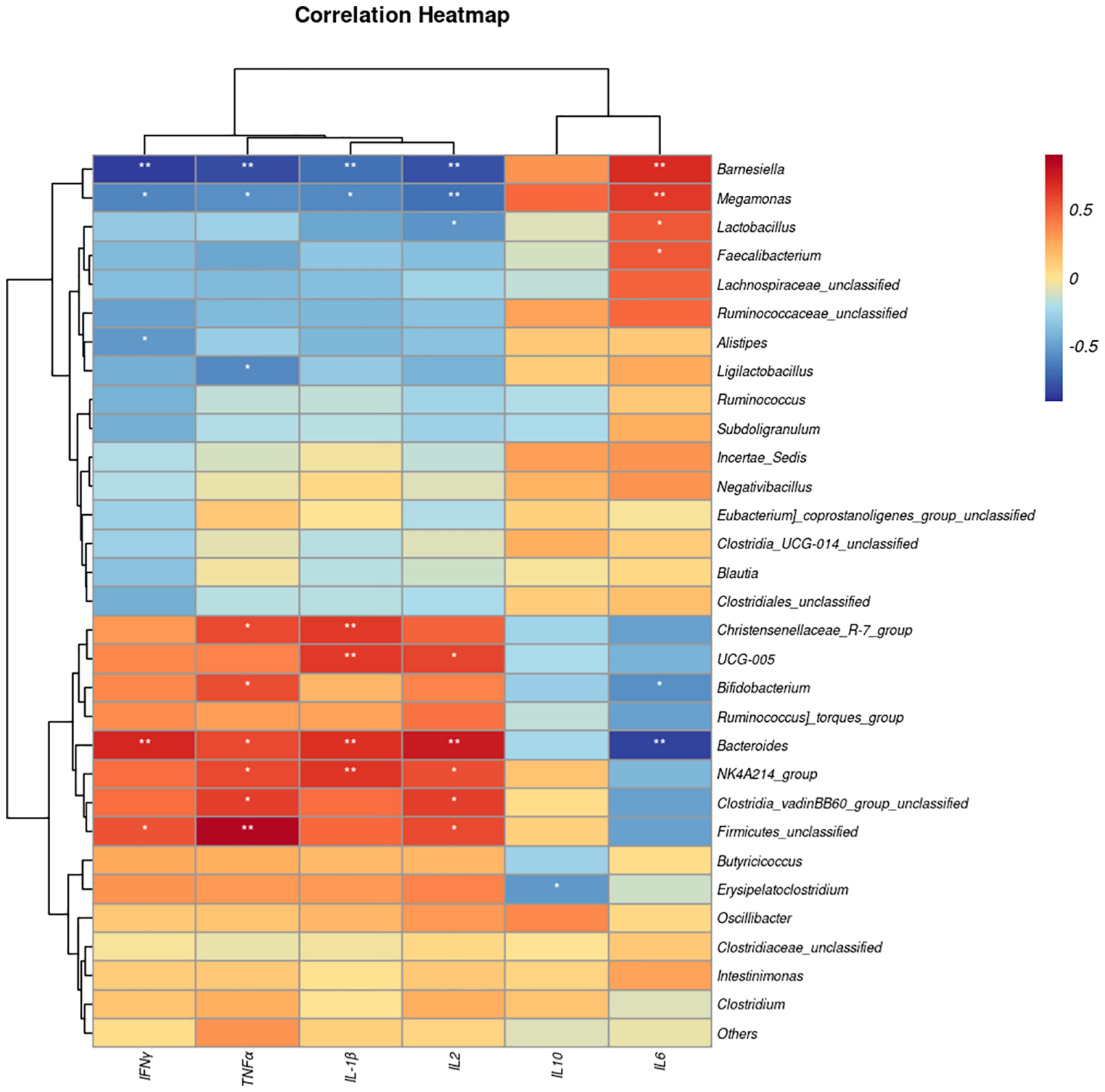
The heat map represents the correlation between serum cytokine levels and caecal microbiota taxa in the EP+YG vs control broiler chickens. *P< 0.05 and **P<0.01. IL-1β, Interleukin 1 beta; IL-2, Interleukin 2; IL-6, Interleukin 6; IL-10, Interleukin 10; TNF-α, Tumor necrosis factor-alpha; IFN interferon-gamma.

We next performed Spearman correlation analyses between the gut microbiota and serum cytokine contents (**Figure 8**). The abundances of *Bacteroides, Clostridia_vadinBB60_group_unclassified, NK4A214_group*, and *Firmicutes_unclassified* had a positive association with IL-2 and TNFα, whereas genera *Barnesiella* and *Magamonas* were negatively associated. In addition, the abundances of *Bacteroides, NK4A214_group, Christensenellaceae_R-7_group* were significantly positively correlated with IL1β, while *Barnesiella* and *Magamonas* were negatively correlated. On the other hand, the relative abundance of *Barnesiella*, *Magamonas*, *Faecalibacterium* and *Lactobacillus* had a significant positive association with IL-6, whereas the abundance of *Bacteroides* and *Bifidobacterium* were negatively associated.

### 3.8 Association analysis between microbiota and SCFA content

**Figure 9:**
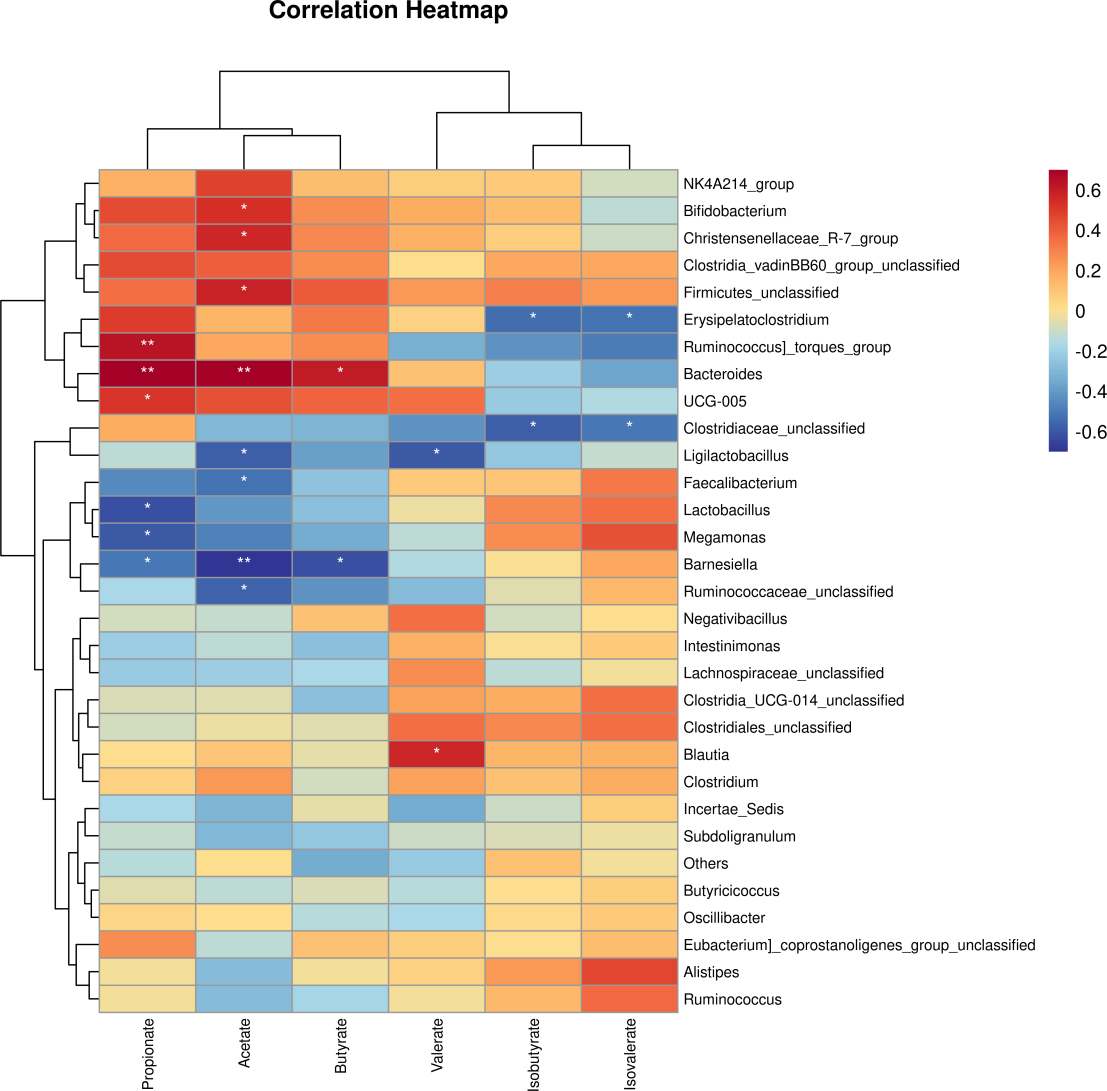
The correlation analysis between caecal microbial genus and short chain volatile fatty acid production in EP+YG treated vs control chickens. *P< 0.05 and **P<0.01.

To investigate whether the change in abundance of bacteria genus had an association with SCFA production, we employed a Spearman correlation analysis between bacteria taxa and SCFAconcentration (**Figure 9**). In this study, the abundance of *Bifidobacterium, Christensenellaceae_R−7_group, Firmicutes_unclassified* and *Bacteroides* had a significant positive association with acetate while *Barnesiella* and *Faecalibacterium* abundance showed a negative association. A significant positive and negative association of the abundance of *Bacteroides* and *Barnesiella with* butyrate production respectively were observed. In addition, *Ruminococcus] _torques_group*, *Bacteroides*, *UGC−005* had a significant positive association with propionate whereas, the abundance of *Lactobacillus, Megamonas, Barnesiella* were negatively associated.

### 3.9 Untargeted metabolomics reveals EP+YG modulates metabolite profiles

**Figure 10:**
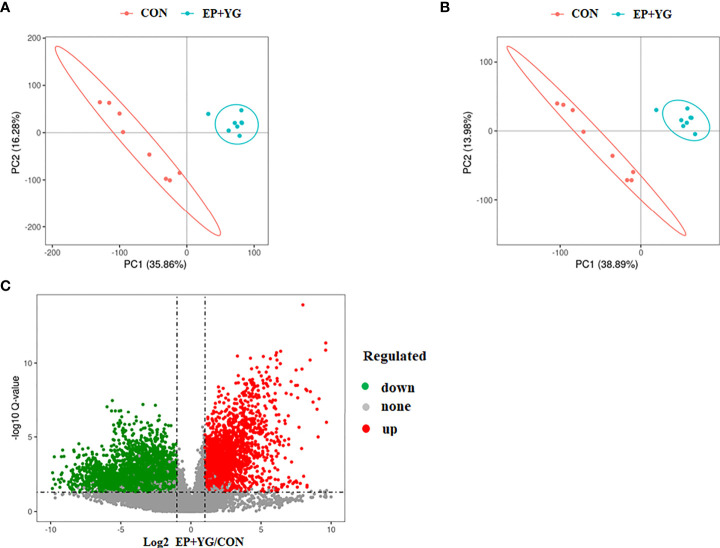
Partial least squares discriminate analysis (PLS-DA) and volcano plot of caecal metabolites in the treatment and control and chickens. The PLS-DA of microbial metabolites in caecal contents **(A)** positive and **(B)** negative ionization mode. **(C)** The volcano plot shows the differential metabolites between treatment groups (red dot: significantly up-regulated metabolites, green dot: significantly down-regulated metabolites, gray dot: the metabolites with no significant difference, *Q* < 0.05). CON (fed basal diet); EP+YG (supplemented with 200 mg EP and 200 mg YG/kg diet in combination).

To assess whether dietary EP and YG supplementation alter the gut metabolites, we detected the caecal content metabolite profiles using untargeted metabolomics. The partial least-squares discriminant analysis (PLS-DA) reflected that supplementation of diet with EP+YG resulted in a distinct separation of identified caecal metabolites from the control group (**Figures 10A, B**).

We identified a total of 13077 spectral features. Based on the criteria of variable importance in the projection (VIP)>1 and the fold change of metabolites less than 0.5 or more than 2, coupling with *P*-value <0.05, 1543 significant differential metabolites in EP+YG vs CON group were identified among these 712 metabolites were upregulated and 831 metabolites were downregulated (**Figure 10C**).

**Figure 11:**
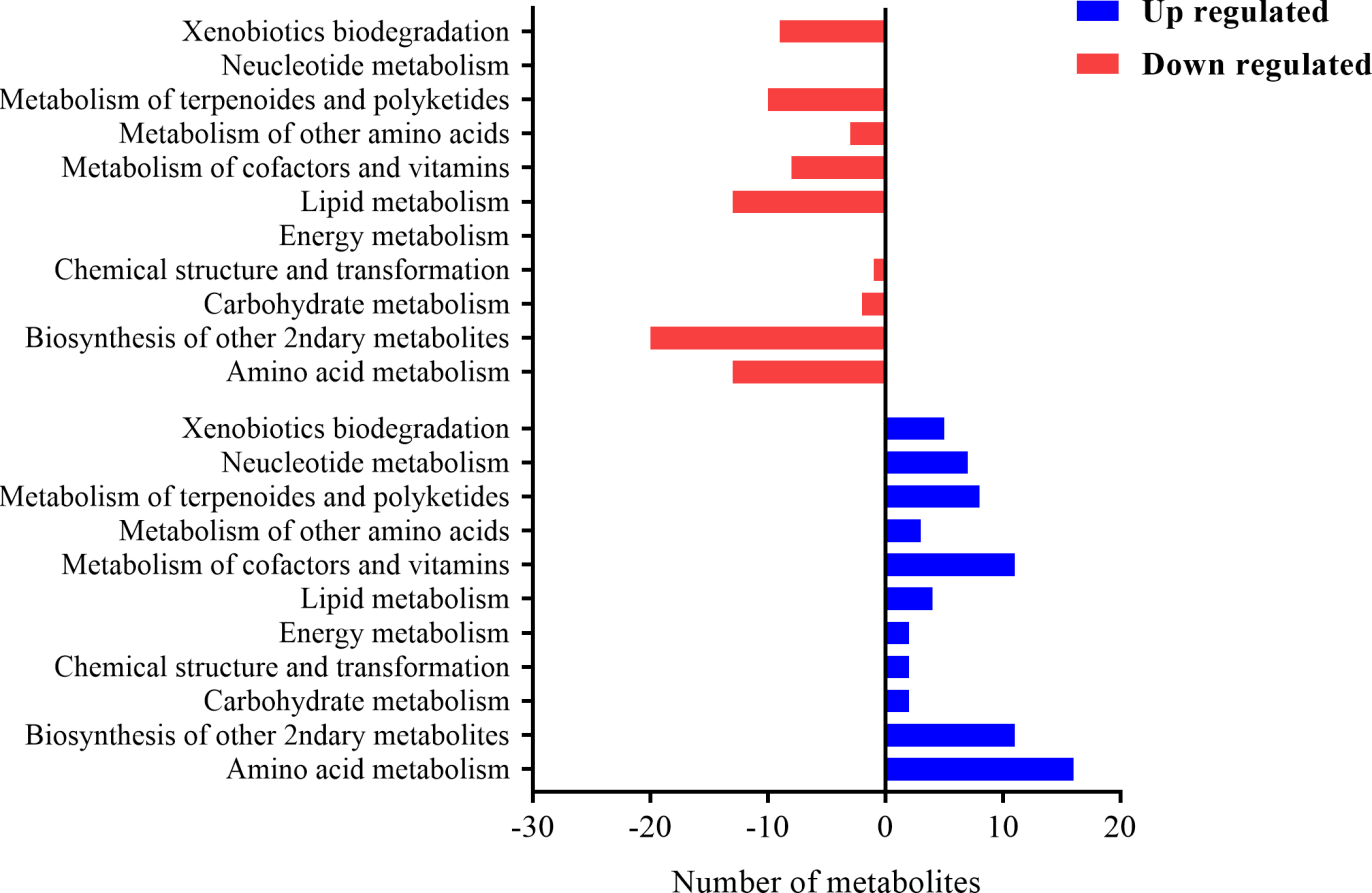
The histogram plot of KEEG pathway analysis of differential metabolites in EP+YG vs control group chickens. The number of metabolites involved in each pathway at the second level of the KEGG pathway.

These metabolites were assigned to the KEGG pathway and 158 metabolites were aligned and enriched in KEEG2, of which 75 were up-regulated and 83 were downregulated. The KEEG results (**Figure 11**) demonstrated that a myriad of metabolites was involved in amino acid metabolism, carbohydrate metabolism, biosynthesis of secondary metabolites, lipid metabolism, and nucleotide metabolism.

**Figure 12:**
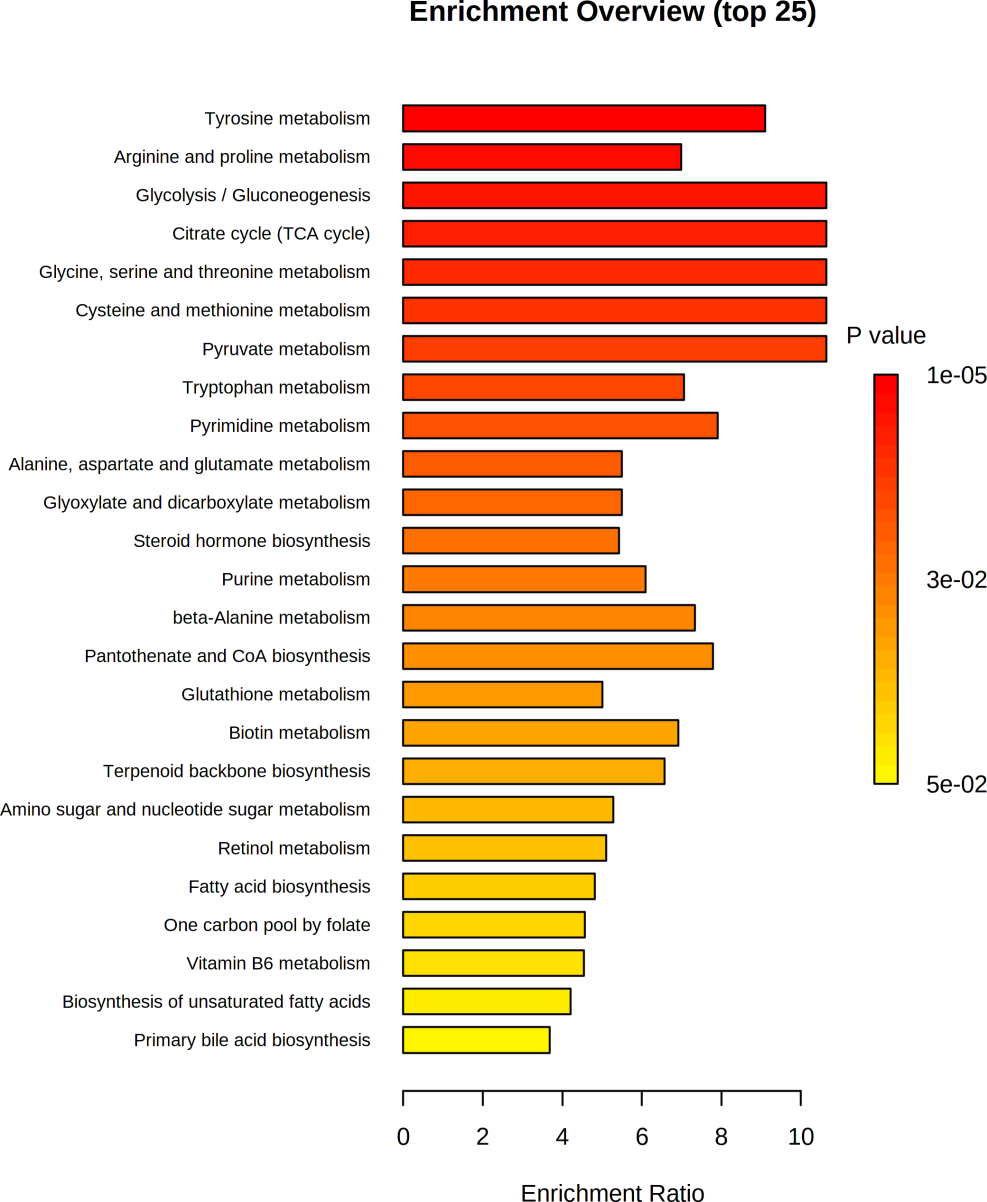
Pathway enrichment analysis of differential metabolites in both positive and negative ionization mode. The Rich Factor in the x-axis indicates the ratio of the number of differential metabolites annotated to the pathway to all the metabolites annotated to the pathway.

We next mapped the metabolic network of altered metabolites using MetaboAnalyst v.5 to identify the important enrichment pathways. The importance of these pathways was ranked based on enrichment ratio and -log(p). The quantitative enrichment analysis results showed that carbohydrate metabolism particularly glycolysis/gluconeogenesis and citric acid cycle, and amino acid metabolism particularly tyrosine metabolism, glycine, serine, and threonine metabolism, and cysteine and methionine metabolism were the most important pathways identified with enrichment ratio >11 (**Figure 12**; **Supplementary Table 2**).

**Figure 13:**
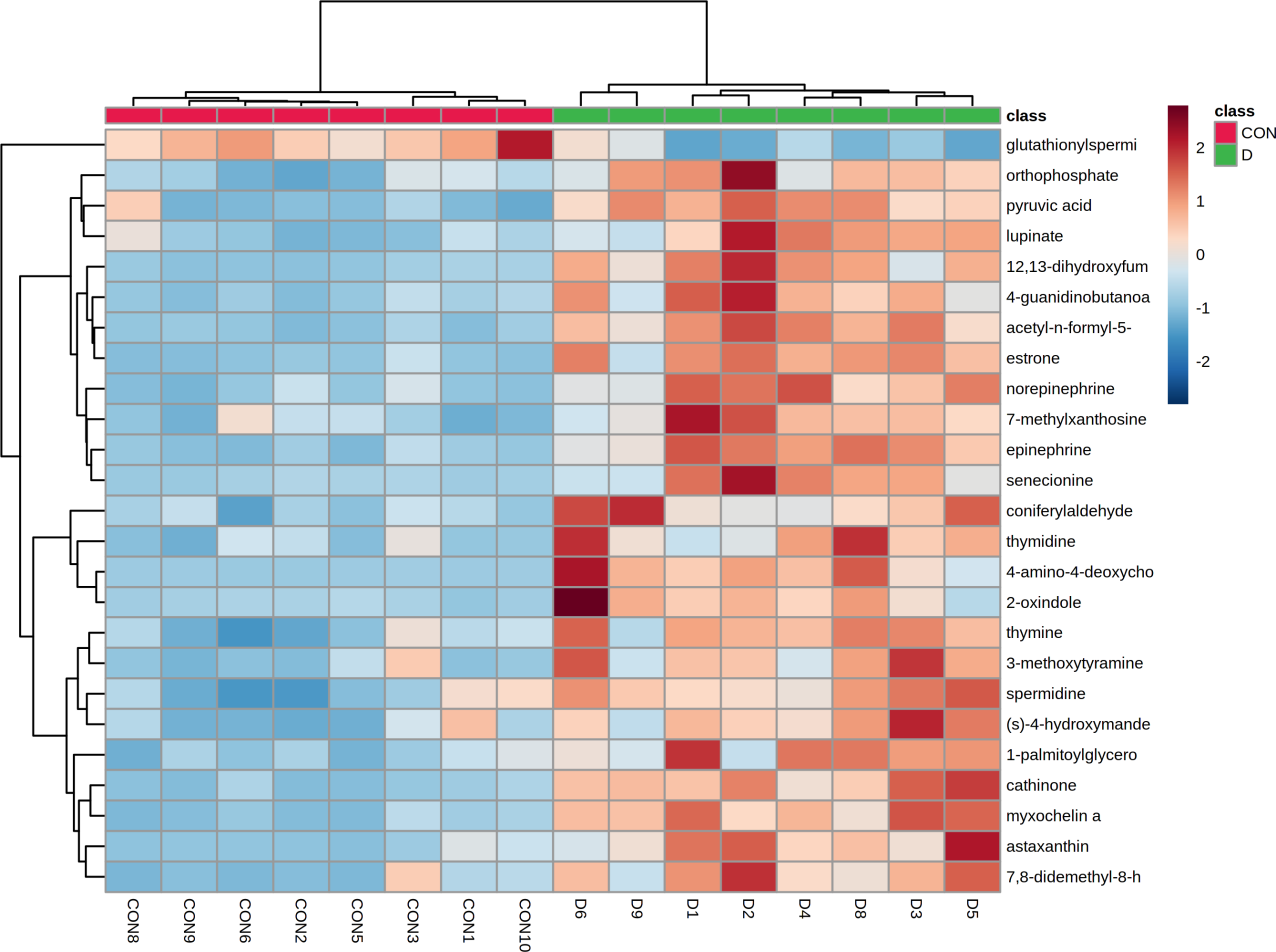
Heat-map of hierarchical clustering analysis of top 30 altered metabolites in the caecal contents of EP+YG vs control groups broiler chickens.

We further performed the hierarchical cluster analysis (HCA) of the differentially accumulated metabolites and found a distinct clustering of metabolites between the two groups (**Figure 13**).

## 4 Discussion

A well-developed adaptive immune system is fundamental to protecting the host from pathogenic microbes and therefore a natural substance that modulates the immune system is a subject of interest. Herein, we supplemented the chicken’s diet with EP+YG and evaluated the immune response by measuring the cytokines content. We found that EP+YG supplementation could induce cytokines markers of IL-2, IL-1β, TNF-α, and IFN-γ in broiler chickens, suggesting that this supplement is a potential immunomodulator. Previous studies have shown that yeast β-glucans could be sensed by the dectin-1 receptor in the immune cells, thereby activating the downstream immune system signaling pathways (36). Similarly, the immune-modulatory effects of EP have been recognized previously *in vivo* and *in vitro* (37).

The role of gut microbiota in the digestion of complex carbohydrate such as polysaccharides have been well established. These microbes live symbiotic with the host and nutritional factors could directly affect their colonization of the gut (38–40). Herein, we did 16S-rRNA sequencing from the caecal content of chickens fed EP and YG in combination to investigate microbiota dysbiosis. We found a non-significant difference between treatment groups in terms of α diversity. Similarly, our previous studies showed that EP supplementation had no significant effect on α-diversity in the caecal contents of chickens (14).

The β-diversity analysis showed that supplementation of EP+YG altered the bacterial community structures evidenced by a distinct clustering with the control group, indicating that it has a potential prebiotic effect that could selectively enrich beneficial microbiota involved in the digestion of a complex carbohydrate diet. The taxonomic analysis at the phylum level further revealed that EP+YG treatment decreased the abundance of *Firmicutes*, *Cyanobacteria*, and *Campylobacterota* and increased *Bacteroidota*, suggesting that EP+YG enriched beneficial bacteria and reduced harmful bacteria phyla. The higher abundance of *Bacteroidota* observed in the treatment group might be due to the presence of many species in this phylum that could participate in the digestion of complex carbohydrates (41, 42). In agreement with the current study, previous studies showed that EP treatment (14, 43), β-glucan (44), and MOS supplementation (45) increased the abundance of *Bacteroidota* and decreased *Firmicutes*.

In agreement with the caecal microbiota dysbiosis at the phylum levels, supplementation of EP+YG could shift microbiota composition at the genus level. EP+YG treatment appeared to increase *Bacteroides*, *Clostridia_vadinBB60_group_unclassified*, *Ruminococcus]_torques_group* and *Bifidobacterium* and decreased *Faecalibacterium*, *Barnesiella*, *Lactobacillus*, *Ligilactobacillus* and *Megamonas*. *Bacteroides* have been reported to ferment diverse types of dietary polysaccharides (46) and are also involved in host immune system development (47), and maintenance of gut microbial balance (48). The *Ruminococcus]_torques_group* was found to involve in gut barrier integrity (49) and positively correlated with the production of proinflammatory cytokines (50). These data suggest that EP+YG supplementation shifts microbiota composition towards the growth of health-promoting gut microbiota. Interestingly, we observed a significant positive association of the abundance of *Bacteroides* and *Clostridia_vadinBB60_group* with SCFA production, suggesting that these bacteria could ferment the EP and YG polysaccharides into useful metabolites. Similarly, a previous study showed that high molecular weight *β*-glucan consumption increased *Bacteroides* abundance in humans (51).

*Bifidobacterium* belongs to Actinobacteria and contains genes involved in the uptake and breakdown of carbohydrates (52). In addition, this bacterium plays an important role in butyrate and acetate short-chain fatty acid production (53). Furthermore, the role of *Bifidobacteria* in innate and adaptive immune responses has been recognized (54). In the present study, EP+YG supplementation increased the abundance of *Bifidobacterium* compared to the control group, suggesting this bacterium may contribute to the fermentation of EP and YG polysaccharides. Furthermore, we found a positive association between the abundance of *Bifidobacteria* with caecal content acetate concentration, which further verifies its role in polysaccharide fermentation. Similarly, a previous study reported an increase in the abundance of *Bifidobacterium* in hens fed MOS (55).

Fat mass and obesity have been associated with type 2 diabetes mellitus (56). Palmas et al. (57) found a positive correlation between *Megamonas* and fat mass. In the present study, we found a reduction in the abundance of *Megamonas* in response to EP+YG supplementation, suggesting that EP+YG reduces harmful bacteria. Previous studies have reported that EP supplementation (58) and yeast β-glucan polysaccharide (59) have an anti-diabetic effect.

Furthermore, in this study, the association between bacterial genus and cytokine production was observed. Some of the bacteria genera such as *Bacteroides, Clostridia_vadinBB60_group_unclassified*, and *Bifidobacterium* had a positive association with proinflammatory cytokine production, suggesting that the immunomodulatory effects of EP+YG may be at least partially through modulating of intestinal microbiota. Consistent with the present study, previous studies showed that EP (13, 14) and yeast β glucan (11) supplementation could regulate gut microbiota.

In addition to structural and community profile change in caecal microbiota, EP+YG supplementation significantly affects the predictive function of microbiota by enriching the functional pathway related to nucleotide metabolism, amino acids metabolism, glycan biosynthesis, and metabolism, energy metabolism, carbohydrate metabolism and biosynthesis of other secondary metabolites, suggesting an important feed additive. Most of the enriched functional pathways observed in the caecal microbiota of EP+YG-supplemented chickens were associated with *Bacteroides*, which have many species involved in SCFA production and immune system modulation (60).

It has become increasingly apparent that the gut microbiome orchestrates the host physiology and immune response through microbial-derived metabolites (61). Thus, a comprehensive understanding of how diet influences the gut microbiota and its metabolites helps to design dietary-based therapies. In this study, nontargeted metabolomics analysis was used to identify whether EP and YG supplementation modulates the caecal metabolites in broiler chickens. The PLS-DA score plots showed distinct clustering of metabolites between treatment and control groups, suggesting that the EP+YG supplementation modulates the metabolic profiles of caecal contents in broiler chickens. This result is consistent with previous studies that showed dietary yeast β-glucan supplementation altered the metabolic profile of chickens (25).

Gut microbiota ferment complex carbohydrates including polysaccharides and produces metabolites such as acetic, propionic, and butyric acids that regulate the various biological process of the body (62). In this study, the concentration of SCFA acetate, butyrate and propionate were higher in the EP+YG group than in the control group. Acetate is produced by intestinal bacteria using pyruvic acid *via* acetyl-CoA (63), which is implicated in host energy homeostasis and immunity (64). Pyruvic acid is a cellular metabolite produced in carbohydrate metabolism. The results of the present study showed that pyruvic acid was upregulated following EP+YG supplementation compared with the control group and further pathway analysis revealed that EP+YG supplementation enriched carbohydrate metabolism, including the glycolysis/gluconeogenesis and citric acid cycle pathways. Therefore, these results suggest that EP+YG supplementation modulates gut microbiota, thereby influencing metabolites production, which is implicated in energy homeostasis and immune response. Our previous studies showed that EP supplementation improved caecal volatile fatty acid content and immune response in chickens (14).

Polysaccharides supplementation has been reported to alter the level of metabolites related to lipid metabolism including oleic acid and docosahexaenoic acid (65). These fatty acids could be synthesized by intestinal microbiota such as *Lactobacillus* species and *Eubacterium ventriosum* from alpha-linoleic acid as a substrate (66). In the present study, differential metabolites associated with fatty acid biosynthesis and biosynthesis of unsaturated fatty acids, such as malonic acid, oleic acid, and docosahexaenoic acid were down-regulated following EP+YG supplementation. Consistently, we observed a reduction in the abundance of *Lactobacillus* species in the caecal contents of the EP+YG supplemented group. Similarly, our previous study showed that EP-Zn supplementation reduced fatty acid profiles in the breast muscle of chicken (15). Thus, the reduction in fatty acid-related metabolites may be associated with the inhibition of microbiota responsible for the biosynthesis of these fatty acids. Furthermore, in this study, an increase in the Acetyl-l-carnitine metabolite was observed in response to EP+YG supplementation. The role of Acetyl-l-carnitine in transferring long‐chain fatty acids into the mitochondria for β‐oxidation have been recognized (67). The data further reinforced the lipid metabolism modulator effects of EP+YG supplementation.

Pyridoxine is a bioactive form of vitamin B-6 deficiency and has antioxidant activity (68) as well as suppressed carcinogenesis by reducing cell proliferation (69). Vitamin B-6, in its coenzyme forms, could involve in nutrient metabolism (70) and is involved in alleviating oxidative stress (71). Different bacterial species such as *Actinobacteria*, *Bacteroidetes*, and *Proteobacteria* have been reported to influence the synthesis of vitamin B-6 (72). A significant increase in the pyridoxine level and enriched vitamin B-6 metabolism in the caecal contents of EP+YG supplemented chickens were observed compared with the control group. These results are consistent with the results obtained in microbiota analysis and microbial function prediction analysis which showed that EP+YG supplementation increased the abundance of *Bacteroides* and enriched vitamin metabolism. Therefore, this study indicated that EP+YG supplementation improved vitamin B-6 metabolism by regulating the intestinal microbiota and their metabolites, particularly by increasing pyridoxine levels, which might have a role in amino acid metabolism, and oxidative stress alleviation and cancer prevention. On the contrary, Zhen et al. (25) observed a significant downregulation in vitamin B-6 metabolism following yeast β-glucan supplementation in older hens. The discrepancy between the current study and the previous study might be the difference in the age of chickens as age is an important factor affecting microbiota composition (73).

Carbohydrate substrate and luminal contents have been shown to regulate amino acid metabolism by modulating the gut microbiota (74). Besides, the gut microbiota could produce various bioactive compounds, including tryptophan, SCFAs, and amines that are involved in the energy metabolism of the host (74). In the present study, we observed a significant increase in spermidine and spermine polyamines in the caecal contents of EP+YG supplemented chickens compared with the control chickens, which are likely to be derived from bacteria (75). Epidemiological data showed that spermidine could reduce cancer development (Fan et al., 2020), alleviate D-galactose-induced oxidative stress (76), and regulate immune response (77). In addition, spermine treatment has been reported to inhibit lipopolysaccharide (LPS)-mediated production of nitric oxide and proinflammatory cytokines, in mouse macrophages (78). This data suggested that the increase in spermidine and spermine contents following EP+YG supplementation might be beneficial for the antioxidant, anticancer, and immune regulation effects of this diet.

Studies have shown that intestinal bacteria could affect neurotransmitters production or generate many neurotransmitters, such as norepinephrine (NE), Dopamine, and 5-hydroxytryptamine (5HT) (79). Dopamine is an important transmitter that has also been reported to modulate immune responses (80) and intestinal inflammation by inhibiting the NLRP3 inflammasome (81). About half of the dopamine is produced in the gastrointestinal tract by intestinal epithelial cells, where microbiota is involved (82). For example, gut microbiota depletion could reduce the synthesis of dopamine in the intestines (83). In the present study, we found a significant increase in the dopamine concentration in the EP+YG group compared with the control group, suggesting that EP+YG could regulate important metabolites that are implicated in neurotransmitter and immune response.

## 5 Conclusion

Generally, the 16S-rRNA sequencing and untargeted metabolomics analysis results demonstrated that EP+YG supplementation altered intestinal microbiota and their metabolites that are implicated in different biological processes. These data suggested that the possible mechanism for the biological activity of EP and YG may be associated with the dynamic shift in microbiota composition and their metabolites in response to these diets. Together, this study suggested the potential prebiotic effects of EP+YG supplementation for broiler chickens.

## Data availability statement

The datasets presented in this study can be found in online repositories. The names of the repository/repositories and accession number(s) can be found in the article/**Supplementary Material**. https://www.ncbi.nlm.nih.gov/bioproject/858221.

## Ethics statement

The animal study was reviewed and approved by Animal Care and Use Committee of the Institute of Subtropical Agriculture, Chinese Academy of Sciences.2021-0036A.

## Author contributions

TW designed the experiment, data collection and analysis, and manuscript writing; BC, TZ, LG data collection and analysis. ZL carried out the animal experiment and sample collection; CX performed the statistical analyses and manuscript revision. XW, design the experiment, manuscript revision and fund acquisition. All authors contributed to the article and approved the submitted version.

## Funding

We would like to acknowledge the earmarked fund for the Key Collaborative Research Program of the Alliance of International Science Organizations (Grant No. ANSO-CR-KP-2021-10), and Taishan industry leading talent blue talent project for their financial support.

## Conflict of interest

The authors declare that the research was conducted in the absence of any commercial or financial relationships that could be construed as a potential conflict of interest.

## Publisher’s note

All claims expressed in this article are solely those of the authors and do not necessarily represent those of their affiliated organizations, or those of the publisher, the editors and the reviewers. Any product that may be evaluated in this article, or claim that may be made by its manufacturer, is not guaranteed or endorsed by the publisher.
