# Supplementary material for: Microbiome-metabolome analysis reveals alterations in the composition and metabolism of caecal microbiota and metabolites with dietary Enteromorpha polysaccharide and Yeast glycoprotein in chickens
Source: Front Immunol. 2022 Oct 13;13:996897. doi: 10.3389/fimmu.2022.996897 (PMC9614668; doi:10.3389/fimmu.2022.996897)
Supplement: Supplementary file 1 [file DataSheet_1.docx]

Supplementary Material

**Supplementary Table 1**. Ingredient composition and nutrient contents of basal diets

| Ingredients, % | 1~21 d | 22~42 d |
| --- | --- | --- |
| Corn | 55.41 | 57.64 |
| Soybean meal, CP43% | 31.00 | 27.30 |
| Corn gluten meal | 5.00 | 5.00 |
| Soybean oil | 3.60 | 5.60 |
| Limestone | 1.20 | 1.20 |
| Dicalcium phosphate | 2.00 | 1.60 |
| _L_-Lysine | 0.34 | 0.25 |
| _DL_-Methionine | 0.15 | 0.11 |
| Premix^1^ | 1.0 | 1.0 |
| Salt | 0.30 | 0.30 |
| Total | 100.0 | 100.0 |
| Nutrient levels, % |  |  |
| Metabolizable energy, MJ/kg | 12.54 | 13.19 |
| Crude protein | 21.09 | 19.60 |
| Calcium | 1.02 | 0.93 |
| Available phosphorus | 0.49 | 0.42 |
| Lysine | 1.20 | 1.06 |
| Methionine | 0.50 | 0.43 |
| Methionine + cysteine | 0.85 | 0.77 |
| Arginine | 1.35 | 1.23 |
| Threonine | 0.80 | 0.74 |

^1^The premix provided per kilogram of diet: vitamin A, 15,600 IU; vitamin D3, 4,480 IU; vitamin E, 31 IU; vitamin B1, 2.4 mg; vitamin B2, 7.2 mg; vitamin B6, 6.3 mg; vitamin B12, 0.32 mg; niacin, 47 mg; pantothenic acid, 16.2 mg; folic acid, 1.6 mg; biotin, 0.26 mg; Cu, 10.4 mg; Fe, 75 mg; Zn, 71 mg; Mn, 83.1 mg; Se, 0.5 mg; I, 0.5 mg.

**Supplementary Table 2**. The quantitative enrichment analysis results of caecal metabolites in EP+YG vs control group broiler chickens.

| Pathway | **Total** | **Hits** | **Statistic** | **Expected** | **P-value** | **Holm P** | **FDR** |
| --- | --- | --- | --- | --- | --- | --- | --- |
| Tyrosine metabolism | 42 | 5 | 60.67 | 6.667 | 1.30E-05 | 4.4E-04 | 2.1E-04 |
| Arginine and proline **metabolism** | 38 | 5 | 46.56 | 6.667 | 1.57E-05 | 5.2E-04 | 2.1E-04 |
| Glycolysis / Gluconeogenesis | 26 | 1 | 70.90 | 6.667 | 4.29E-05 | 1.4E-03 | 2.1E-04 |
| Citrate cycle (TCA cycle) | 20 | 1 | 70.90 | 6.667 | 4.29E-05 | 1.4E-03 | 2.1E-04 |
| Glycine, serine and threonine metabolism | 33 | 1 | 70.90 | 6.667 | 4.29E-05 | 1.4E-03 | 2.1E-04 |
| Cysteine and methionine metabolism | 33 | 1 | 70.90 | 6.667 | 4.29E-05 | 1.4E-03 | 2.1E-04 |
| Pyruvate metabolism | 22 | 1 | 70.90 | 6.667 | 4.29E-05 | 1.4E-03 | 2.1E-04 |
| Tryptophan metabolism | 41 | 3 | 47.07 | 6.667 | 1.40E-04 | 3.8E-03 | 6.0E-04 |
| Pyrimidine metabolism | 39 | 4 | 52.70 | 6.667 | 2.87E-04 | 7.4E-03 | 1.1E-03 |
| Alanine, aspartate and glutamate metabolism | 28 | 2 | 36.61 | 6.667 | 5.58E-04 | 1.4E-02 | 1.7E-03 |
| Glyoxylate and dicarboxylate metabolism | 32 | 2 | 36.61 | 6.667 | 5.58E-04 | 1.4E-02 | 1.7E-03 |
| Steroid hormone biosynthesis | 85 | 4 | 36.14 | 6.667 | 9.69E-04 | 2.2E-02 | 2.6E-03 |
| Purine metabolism | 65 | 3 | 40.65 | 6.667 | 9.81E-04 | 2.2E-02 | 2.6E-03 |
| beta-Alanine metabolism | 21 | 2 | 48.87 | 6.667 | 1.40E-03 | 2.9E-02 | 3.4E-03 |
| Pantothenate and CoA biosynthesis | 19 | 1 | 51.91 | 6.667 | 1.64E-03 | 3.3E-02 | 3.7E-03 |
| Glutathione metabolism | 28 | 3 | 33.36 | 6.667 | 1.81E-03 | 3.4E-02 | 3.8E-03 |
| Biotin metabolism | 10 | 1 | 46.08 | 6.667 | 3.83E-03 | 6.9E-02 | 7.7E-03 |
| Terpenoid backbone biosynthesis | 18 | 1 | 43.79 | 6.667 | 5.24E-03 | 8.9E-02 | 9.9E-03 |
| Amino sugar and nucleotide sugar metabolism | 37 | 1 | 35.21 | 6.667 | 1.54E-02 | 2.5E-01 | 2.8E-02 |
| Retinol metabolism | 17 | 2 | 34.06 | 6.667 | 1.76E-02 | 2.6E-01 | 3.0E-02 |
| Fatty acid biosynthesis | 47 | 1 | 32.12 | 6.667 | 2.21E-02 | 3.1E-01 | 3.6E-02 |
| One carbon pool by folate | 9 | 1 | 30.44 | 6.667 | 2.67E-02 | 3.5E-01 | 4.0E-02 |
| Vitamin B6 metabolism | 9 | 1 | 30.30 | 6.667 | 2.71E-02 | 3.5E-01 | 4.0E-02 |
| Biosynthesis of unsaturated fatty acids | 36 | 2 | 28.04 | 6.667 | 2.96E-02 | 3.5E-01 | 4.2E-02 |
| Primary bile acid biosynthesis | 46 | 1 | 24.58 | 6.667 | 5.08E-02 | 5.1E-01 | 6.9E-02 |
